# Association between the safety climate and occupational injury in the Korean working population: a cross-sectional study

**DOI:** 10.4178/epih.e2024082

**Published:** 2024-10-01

**Authors:** Jeehee Min, Tae-Won Jang, Hye-Eun Lee, Mo-Yeol Kang, Seong-Sik Cho

**Affiliations:** 1 Department of Occupational and Environmental Medicine, Hanyang University Hospital, Seoul, Korea; 2 Department of Occupational and Environmental Medicine, Hanyang University College of Medicine, Seoul, Korea; 3 Department of Social and Preventive Medicine, Hallym University College of Medicine, Chuncheon, Korea; 4 Department of Occupational and Environmental Medicine, Seoul St. Mary’s Hospital, College of Medicine, The Catholic University of Korea, Seoul, Korea; 5 Department of Occupational and Environmental Medicine, Dong-A University College of Medicine, Busan, Korea

**Keywords:** Safety climate, Occupational injuries, Safety management, Organizational culture

## Abstract

**OBJECTIVES:**

Preventing occupational injuries remains a significant challenge in Korea. A positive safety climate can contribute to reducing workplace injuries. However, the impact of safety climate on preventing occupational injuries among the Korean workforce has not been adequately explored. Therefore, this study aimed to investigate the relationship between the perceived safety climate and occupational injuries within the Korean working population.

**METHODS:**

This study used baseline data from the Korean Work, Sleep, and Health Study (KWSH). The safety climate was measured using the brief version of the Nordic Safety Climate Questionnaire. Occupational injury was determined by whether injuries or accidents had occurred at workplaces in the past year. Logistic regression analysis was performed to examine the association between the safety climate and occupational injury.

**RESULTS:**

Participants who reported an unfavorable workplace safety climate were more likely to experience occupational injuries. Multiple logistic regression analysis revealed that the adjusted odds ratio (OR) for occupational injuries in an unfavorable safety climate was 2.20 (95% confidence interval [CI], 1.38 to 3.51) compared to a favorable safety climate. Specifically, factors such as “not encouraging employees to follow safety rules when on a tight schedule” (OR, 2.02; 95% CI, 1.25 to 3.24) and “not helping each other work safely” (OR, 1.98; 95% CI, 1.17 to 3.25) were significantly associated with occupational injuries.

**CONCLUSIONS:**

An unfavorable safety climate was associated with increased occupational injuries among Korean workers. Improving the safety climate in the workplace may reduce occupational injuries in Korea.

## GRAPHICAL ABSTRACT

[Fig f1-epih-46-e2024082]

## Key Message

Safety climate was related to occupational injuries in the Korean working population. This result may indicate that occupational injuries can be prevented by improving the workplace safety climate. Workplace safety should be a high priority goal for management, and workers need to help each other for workplace safety.

## INTRODUCTION

Occupational injuries continue to be a significant concern in Korea. Although the incidence of these injuries is on the decline, there were still 875 fatalities due to severe occupational injuries in 2022. Many workplaces in Korea tend to avoid reporting such injuries, preferring this to strengthening safety measures and adopting practices that could prevent them [1,2]. This tendency to underreport is often driven by a desire to evade punitive actions and reduce compensation insurance costs [3,4]. When examining occupational injuries in Korea, it is important to consider the likelihood of underreporting. The current worker’s compensation system in Korea is generally restricted to those covered by workers’ compensation insurance, which likely contributes to the underreporting of occupational accidents. Furthermore, workers who are part of racial or ethnic minority groups, or those in temporary positions, may hesitate to report injuries due to concerns about adverse effects on their employment prospects, including opportunities for re-employment and promotion [1].

Preventing occupational injuries necessitates a multi-layered approach involving various social efforts. Governments are responsible for enacting appropriate legal regulations and conducting thorough labor inspections. It is imperative for each workplace to adhere to these governmental regulations to maintain safe and healthy working conditions. Moreover, prioritizing the creation of a safe and healthy workplace environment should be a fundamental aspect of corporate management objectives. Additionally, strengthening the safety culture at workplaces is crucial to enhancing the adoption of safety practices throughout all work processes [5,6].

The Serious Accident Punishment Act was recently introduced in Korea. Despite the existing legal framework for workplace safety being inadequate, this act signifies an effort to enhance safety measures. Moreover, enhancing workplace safety could lead to a reduction in both fatal and non-fatal workplace injuries [7,8]. Beyond governmental regulations, it is crucial to strengthen the safety culture within workplaces, ensuring that safety becomes a top priority for corporate management [4,9]. Furthermore, adequate resources, including safety personnel, must be allocated to establish safe working environments [10-12].

Safety culture comprises the collective values, attitudes, perceptions, competencies, and behavioral patterns that individuals and groups exhibit within an organizational context [13]. It influences the level of commitment, approach, and effectiveness of health and safety practices. Safety culture is generally stable and persists over time. In contrast, safety climate reflects employees’ perceptions of the current state of safety in the workplace, serving as a “snapshot” of the broader safety culture [14]. The primary difference between safety culture and safety climate lies in the assessment challenges. safety culture is difficult to directly evaluate due to its foundation in deeply ingrained ideas and values [15], whereas safety climate is easier to assess because it is related to current attitudes and commitments, which can be assessed using quantitative tools such as questionnaires [15,16].

Safety climate refers to employees’ current perceptions of the importance of safe conduct within their organizational practices, procedures, and behaviors [13], reflecting how employees perceive their organization’s current safety culture [10,11,15,17-23]. Organizations that prioritize and cultivate a positive safety climate generally experience better safety outcomes, higher employee morale, and greater benefits compared to those that do not [11,13,21- 23]. The concept of safety climate is considered indicative of safety-related outcomes, especially in relation to occupational injuries [24]. Moreover, an enhanced safety climate can lead to increased workplace productivity, potential financial gains, and improvements in employee health and safety. Zohar et al. [13] assessed safety climate by measuring organizational responsibility, safety supervision, workers’ safety attitudes, and company safety precautions.

Several studies have analyzed safety climates and their relationship with the work environment in Korea [24-30]. However, the role of the safety climate in preventing occupational injuries in Korea has not been sufficiently elucidated [24,28,31]. Empirical studies on the association between safety climate and occupational injuries are scarce. Therefore, this study explored the relationship between the perceived safety climate and occupational injuries among the Korean working population.

## MATERIALS AND METHODS

### Study population

This cross-sectional study utilized baseline data from the Korean Work, Sleep, and Health Study, a longitudinal study that began in August 2022 [32,33]. This study was launched to explore the working conditions, sleep patterns, and health of Korean workers. Data collection was conducted via an Internet platform, gathering information on sleep, chronotype, socioeconomic status, physical and psychosocial work environments, and health status through questionnaires. The survey was carried out by Embrain, a company specializing in online panel studies, using a convenience sample. Participants were selected to mirror the general working population of Korea in terms of age, sex, and occupational distribution. Those who agreed to participate after receiving an online recruitment letter were required to complete a screening questionnaire. Eligible participants (waged employees) were then sent the study details and an online consent form. Although the survey was conducted online and may not have been fully representative of the entire Korean workforce, the study population was defined as Korean workers who participated in this online survey. Initially, 5,517 participants were selected; however, 5 farmers were subsequently excluded from the analysis. Thus, the study included a total of 5,512 participants.

### Safety climate

The safety climate was assessed using the brief version of the Nordic Safety Climate Questionnaire (NOSACQ)-50. This brief version comprises 5 items and was also included in the Danish Working Environment and Health Cohort Study. Developed in 2010, the NOSACQ-50 is a validated and reliable tool for assessing an organization’s safety climate [16]. However, the Korean language version of the NOSACQ-50 has not yet been validated. We employed the same 5-question questionnaire about safety climate used in the Danish Working Environment and Health Cohort Study [17]. The Korean language version of the NOSACQ-50 should be validated and more broadly utilized in the future. This comprehensive safety climate measurement tool has been validated in over 45 languages, including various European and Asian languages [34]. It includes 7 domains: (1) management’s safety prioritization, commitment, and competence; (2) management’s safety empowerment; (3) management’s safety justice; (4) workers’ safety commitment; (5) workers’ safety priority and non-acceptance of risk; (6) safety communication, learning, and trust in coworkers’ safety competence; (7) trust in the efficacy of safety systems.

The “management’s safety priority, commitment, and competence” concepts in the NOSACQ-50 were assessed using the following 3 statements: (1) management ensures that everyone receives the necessary information on safety, (2) management encourages employees to follow safety rules even when the work schedule is tight, and (3) management involves employees in decisions regarding safety. Workers’ safety commitment was measured using 2 statements: (4) employees help each other work safely and (5) employees consider minor accidents a normal part of daily work (negated or reversed item). Respondents were asked to answer these 5 questions by selecting “agree,” “strongly agree,” “disagree,” or “strongly disagree.” Responding with “strongly disagree” or “disagree” to questions 1 to 4 and “strongly agree” or “agree” to question 5 was considered indicative of problems with the safety climate of the workplace. Overall, the safety climate was classified into 2 categories: favorable and unfavorable. Two or more unfavorable responses were regarded as indicative of an unfavorable safety climate, whereas 1 or no unfavorable response was considered indicative of a favorable safety climate.

### Occupational injuries

The question used to assess occupational injuries was: “In the past 12 months, have you experienced any safety accidents or injuries, including toxic substance poisoning and choking accidents?” The response options provided were “yes” or “no.”

### Occupations and other covariates

Occupations were divided into 4 groups: managers and professionals; office workers; service and sales workers; and manual workers. This classification is a reclassification of occupational categories to align with the International Standard Classification of Occupation (ISCO). The managers and professionals group included employees in senior management positions (e.g., bank branch manager, director of a general company, director of a government department, high-level government officials, judges, prosecutors) and professionals (e.g., lawyers, doctors, researchers, paralegals, pastors, teachers). This grouping corresponds to the ISCO classification, which combines Major Group 1: Legislators, Senior Officials, and Managers; Major Group 2: Professionals; and Major Group 3: Technicians and Associate Professionals. Office workers were defined as those employed in general companies, public enterprises, or government offices, working under the direction of senior management. Service and sales workers included individuals in sales roles (e.g., sales managers, store owners, store sellers, insurance agents, retailers, wholesalers, store cashiers) and service occupations (e.g., caregivers, chefs or cooks, restaurant workers, hairdressers, delivery personnel). This classification follows the ISCO structure, which merges Major Group 4: Clerks with Major Group 5: Service Workers and Shop and Market Sales Workers. Manual workers comprised individuals performing skilled labor (e.g., auto mechanics, painters, electricians, machinists, mechanics), semi-skilled labor (e.g., bricklayers, bus drivers, carpenters, metal workers, bakers), or unskilled labor (e.g., manual laborers, porters, and other blue-collar workers). This classification adheres to the ISCO, which integrates Major Group 7: Craft and Related Trades Workers; Major Group 8: Plant and Machine Operators and Assemblers; and Major Group 9: Elementary Occupations. We excluded farmers and fishery workers, classified under Major Group 6: Skilled Agricultural and Fishery Workers.

Sex, age, educational level, income, employment status, working hours, shift work, presence of labor unions, and presence of workplace safety organizations were included as covariates in this study. Age was categorized into 5 groups: 20-29, 30-39, 40-49, 50-59, and ≥ 60 years. Educational level was divided into 2 categories: high school or lower, and college or higher. Income was segmented into 5 groups based on the respondents’ monthly earnings. Employment status was classified as either direct or indirect. Direct employment referred to individuals who were hired directly by the company, while indirect employment encompassed workers hired by subcontractors, freelancers, and self-employed individuals. Shift work was defined as working hours outside the standard daytime period of 9 a.m. to 6 p.m.

### Statistical analysis

Demographic characteristics of participants who perceived their workplace safety climate as favorable were compared with those who viewed it as unfavorable. We also compared occupational conditions, working hours, shift work, type of occupation, and the presence of labor unions and workplace safety organizations across different safety climate categories. The chi-square test was utilized to assess the relationship between safety climate categories and specific safety issues, based on the participants’ experiences with occupational injuries. Logistic regression analysis was employed to calculate the odds ratios (ORs) and 95% confidence intervals (CIs) for occupational injuries. We performed multiple logistic regression analysis, adjusting for variables such as sex, family income, working hours, and the presence of labor unions and workplace safety organizations. Additionally, a subgroup analysis was conducted focusing on the presence of labor unions. All statistical analyses were carried out using the R version 4.2.2 (R Foundation for Statistical Computing, Vienna, Austria).

### Ethics statement

The study’s research protocol received approval from the Dong-A Institutional Review Board (2-1040709-AB-N-01-202202-HR-017-12). All participants provided informed consent.

## RESULTS

The participant’s general characteristics are outlined in Table 1. Of the 5,512 participants analyzed, 34.0% (n=1,875) indicated that their workplaces had an unfavorable safety climate. Furthermore, 33.5% of male participants reported an unfavorable safety climate at their workplaces. Notably, in the age group of 30-39 years, participants with at least a college education, those with direct contract employment, lower-income employees, and individuals working more than 52 hr/wk tended to report a poorer safety climate. Among the various occupational groups, sales and service workers most frequently reported an unfavorable safety climate. Participants employed in organizations lacking labor unions or safety health governance were also more likely to report a more unfavorable safety climate (Table 1).

The details of occupational injuries in the study population and related factors are presented in Table 2. Seventy-seven participants (1.4%) experienced occupational injuries within the past year. The incidence of occupational injuries was higher among males and those aged 30-39 years than among females and participants in other age groups. Factors such as indirect employment, long working hours, and shift work were linked to a higher incidence of work-related injuries. Interestingly, the presence of labor unions and safety organizations was also associated with occupational injuries (Table 2).

Table 3 illustrates the relationship between safety climate and occupational injuries. The incidence of occupational injuries (2.0%) was higher in an unfavorable safety climate compared to a favorable one. Concerning the questionnaire items on safety climate, the frequency of occupational injuries varied significantly. Specifically, the questions “Management encourages employees to follow safety rules even when the work schedule is tight” and “Employees do not help each other to work safely” were both significantly associated with a higher number of occupational injuries.

The logistic regression analysis showed that an unfavorable safety climate was significantly associated with occupational injury after adjusting for sex (OR, 1.93; 95% CI, 1.22 to 3.04) (Table 4). This significant association persisted even after further adjustments for family income, working hours, occupation, and employment status in model 2 (OR, 1.91; 95% CI, 1.21 to 3.02). The consistency of the association was maintained after additional adjustments for the presence of labor unions and workplace safety organizations (OR, 2.20; 95% CI, 1.38 to 3.51). The relationship between individual questionnaire items on safety climate and occupational injury was also analyzed. Specifically, management’s failure to enforce strict adherence to safety rules during tight work schedules was significantly associated with occupational injuries (model 1: OR, 1.79; 95% CI, 1.11 to 2.82; model 2: OR, 1.75; 95% CI, 1.09 to 2.79; model 3: OR, 2.02; 95% CI, 1.25 to 3.24). Additionally, the lack of mutual assistance among employees in maintaining safety was linked to occupational injuries (model 1: OR, 1.80; 95% CI, 1.08 to 2.93; model 2: OR, 1.77; 95% CI, 1.05 to 2.88; model 3: OR, 1.98; 95% CI, 1.17 to 3.25).

The safety climate was analyzed based on the presence of labor unions (Supplementary Material 1). Among participants employed in companies with a union, 19.0% reported an unfavorable safety climate, compared to 28.8% in companies without a union. We evaluated the relationship between occupational injury and safety climate, along with its components, stratified by occupation into white-collar, blue-collar, and pink-collar groups (Supplementary Material 2). A statistically significant association between an unfavorable safety climate and higher rates of occupational injuries was observed only in the white-collar group.

## DISCUSSION

This study analyzed the association between safety climate and occupational injuries. An unfavorable safety climate was linked to an increased risk of occupational injuries, even after adjusting for potential confounders. The commitment of workplace management to prioritize safety, along with workers’ cooperation in maintaining safety, was significantly associated with the occurrence of occupational injuries. Furthermore, the findings underscore the importance of emphasizing cooperation among workers to improve workplace safety.

A crucial aspect of the safety climate is the role of management, which should prioritize safety [12,35]. However, in most organizations, management considers productivity more important than safety. In the United States, 38% of young farm workers reported that their managers or supervisors were primarily interested in the speed of task completion, showing little concern for safety [23]. A positive safety climate enhances the value placed on safety within an organization [28]. Research has shown that the safety climate is a key determinant of both safety performance and the occurrence of occupational injuries [11,36]. Additionally, the safety climate influences safety training, a fundamental aspect of safety management practices [37]. In Korea, companies with more than 5 employees are required to provide safety training [38]. Employees in hazardous environments, such as construction sites, must undergo more extensive training [38]. Safety training functions as a mediator between safety performance and motivation [37,39]. However, knowledge of safety skills is considered generic and less critical than active participation in safety protocols [40]. A study of restaurant employees demonstrated that management’s commitment significantly influences employees’ perceptions of safety training, underscoring the importance of management’s role in promoting a favorable safety climate [35].

Workers’ cooperation in adopting safety measures and addressing minor accidents plays a crucial role in preventing workplace injuries. This study underscores the importance of such cooperation for injury prevention [19,41,42]. A related study on the safety climate among nurses in the United States found that teamwork and working conditions were inversely related to patient safety incidents and occupational injuries [42]. Furthermore, helping coworkers adhere to safety practices is essential for fostering a commitment to these measures [19,37,41]. Although this study did not find a direct association between neglecting minor accidents and occupational injuries, overlooking near misses and minor incidents could lead to their recurrence or even to more severe accidents in the future [28]. Organizational safety policies that offer personal or group incentives or impose penalties for specific behaviors may result in the underreporting of occupational injuries [1-3,19,41]. Therefore, meticulous reporting of near misses and addressing minor accidents are critical preventive strategies against occupational injuries.

This study investigated the role of labor unions in preventing occupational injuries and found that their presence was associated with a favorable safety climate. Numerous studies have shown that both labor unions and safety organizations can improve the safety climate at workplaces [43,44]. The findings from these studies, along with the results of the current study, suggest that labor unions and safety organizations can be effective in preventing occupational injuries.

A higher incidence of occupational injuries was observed in groups with labor union representation. Notably, the injuries in this study were based on self-reported data, rather than on administratively filed or approved injury claims. It is possible that unionized workplaces have larger, more hazardous environments. However, it is unclear how the presence of a union correlates with the level of workplace danger. Additionally, the severity of injuries could not be assessed with this data. These limitations might account for the surprisingly small difference in injury rates between manual and white-collar workers, and the counterintuitive finding of higher rates among white-collar workers.

The Serious Accident Punishment Act of Korea, enacted in 2022, mandates employer responsibility in safety accidents [45]. Although this act may improve safety in workplaces, simple regulations alone are not enough to sustain a positive safety culture. A comprehensive approach is essential to prevent both fatal and non-fatal workplace injuries [7,8]. To achieve this, improvements in legal regulations, workplace safety culture, and commitment are necessary. The safety climate is quantifiable and can be used to monitor improvements or declines in workplace safety culture. Tools like NOSACQ provide components that clearly define practical and detailed roles, ensuring that both management and workers are dedicated to promoting a positive safety culture [11,13,19]. Additionally, the concept of a safe climate should be broadly embraced, and the safety climate in workplaces should be regularly assessed to enhance safety in Korea. By improving the safety climate, we can reduce the incidence of fatal and non-fatal workplace injuries.

This study has some limitations. First, as over half of the participants were white-collar workers, we cannot exclude the possibility of selection bias. To address this, we performed a stratification analysis by occupation, but no statistically significant findings emerged for the groups of sales and service workers or manual laborers. This may be attributed to the limited number of outcome variables, such as occupational injuries, analyzed in this study. Second, this cross-sectional study does not allow for the assessment of temporal differences between the participants’ perceived safety climate and their experiences with occupational injuries over the past 12 months. Consequently, we cannot establish a temporal relationship. Third, the Korean language version of the Nordic safety climate questionnaire has not yet been validated. However, the 5 questions utilized in this study are essential elements of the workplace safety climate, transcending cultural and linguistic differences. This is supported by the validation of other Asian language versions of the NOSAQ, including those in Japanese and Vietnamese [34]. Additionally, the incidence of occupational injury was determined using a self-reported questionnaire rather than objective data sources such as administrative records or compensation claims. Therefore, the unique aspects of Korean culture and language likely had a minimal impact on the study’s findings. Finally, the study only analyzed the general safety climate of workers, making it difficult to assess the impact of specific work characteristics of the participants. Further research is needed to explore the safety climate and workplace accidents, particularly focusing on the job characteristics of sales and service workers, which are less frequently reported compared to those of manual workers.

The study identified an association between safety climate and the incidence of injuries among Korean workers, utilizing a portion of the NOSACQ-50 to facilitate international comparisons. To prevent occupational injuries, it is crucial to prioritize workplace safety and emphasize the importance of safety cooperation among workers.

This study demonstrated an association between the safety climate and occupational injuries among Korean workers. Both management’s commitment to safety and cooperation among workers were linked to a lower incidence of occupational injuries. Therefore, workplace management should prioritize safety and enhance the safety climate to reduce work-related injuries.

## Figures and Tables

**Figure f1-epih-46-e2024082:**
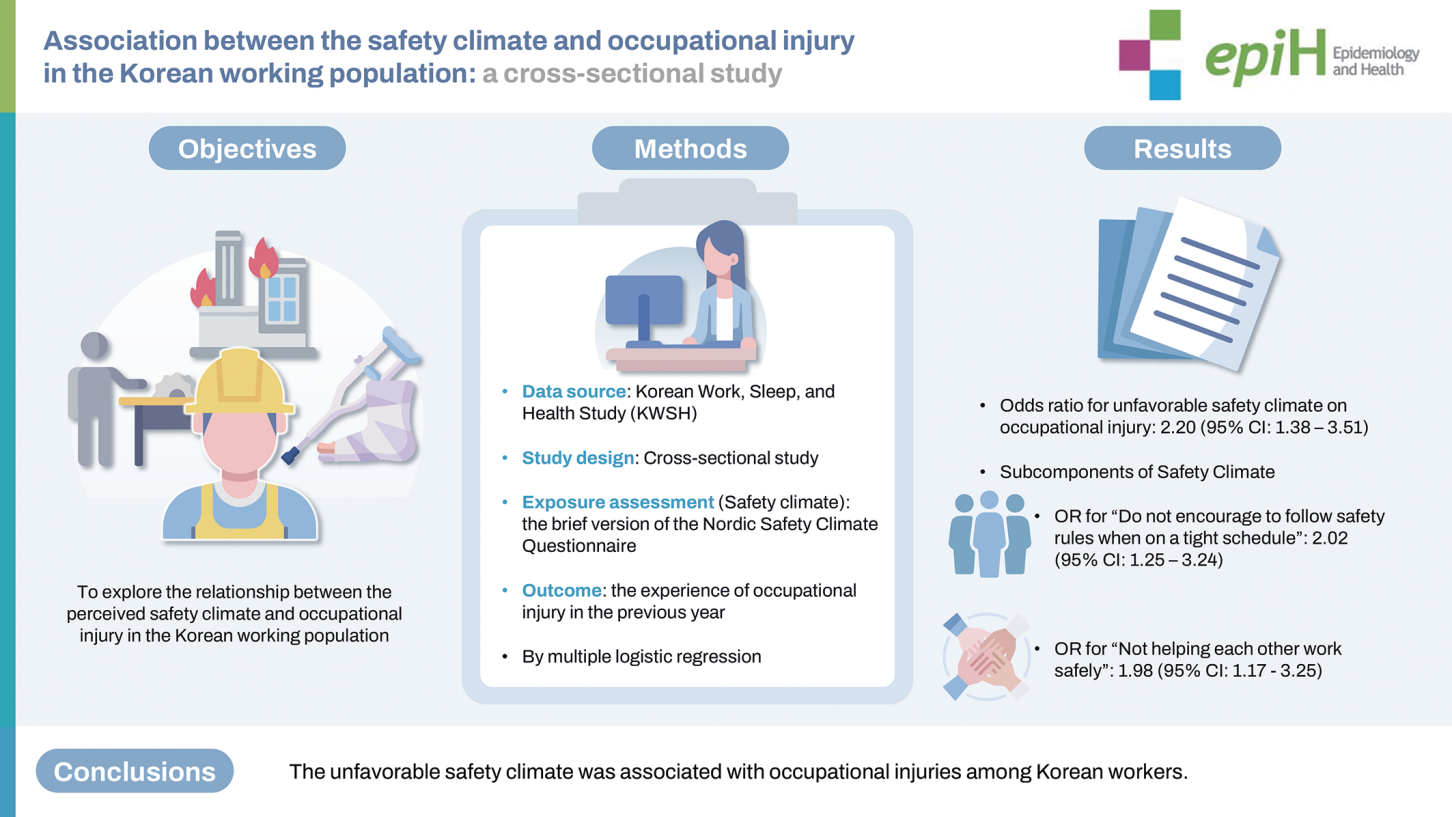


**Table 1.** General characteristics of the study population

Characteristics	Total	Favorable safety climate (<2 safety issues)	Unfavorable safety climate (≥2 safety issues)
Total	5,512 (100)	3,637 (66.0)	1,875 (34.0)
Sex			
Male	3,002 (54.5)	1,997 (66.5)	1,005 (33.5)
Female	2,510 (45.5)	1,640 (65.3)	870 (34.7)
Age (yr)			
20-29	1,059 (19.2)	678 (64.0)	381 (36.0)
30-39	1,068 (19.4)	649 (60.8)	419 (39.2)
40-49	1,306 (23.7)	805 (61.6)	501 (38.4)
50-59	1,393 (25.3)	983 (70.6)	410 (29.4)
≥60	686 (12.4)	522 (76.1)	164 (23.9)
Education			
High school or lower	860 (15.6)	593 (69.0)	267 (31.0)
College or higher	4,652 (84.4)	3,044 (65.4)	1,608 (34.6)
Employment			
Direct	4,894 (88.8)	3,220 (65.8)	1,674 (34.2)
Indirect	618 (11.2)	417 (67.5)	201 (32.5)
Income			
Q1 (lowest)	631 (11.4)	409 (64.8)	222 (35.2)
Q2	3,204 (58.1)	2,052 (64.0)	1,152 (36.0)
Q3	1,079 (19.6)	734 (68.0)	345 (32.0)
Q4	509 (9.2)	368 (72.3)	141 (27.7)
Q5 (highest)	89 (1.6)	74 (83.1)	15 (16.9)
Working hours (hr/wk)			
<40	3,554 (64.5)	2,411 (67.8)	1,143 (32.2)
41-52	1,624 (29.5)	1,040 (64.0)	584 (36.0)
>52	334 (6.1)	186 (55.7)	148 (44.3)
Shiftwork			
Day work	4,945 (89.7)	3,268 (66.1)	1,677 (33.9)
Shiftwork	567 (10.3)	369 (65.1)	198 (34.9)
Occupation			
Managerial and professional	641 (11.6)	456 (71.1)	185 (28.9)
Office worker	3,088 (56.0)	2,049 (66.4)	1,039 (33.6)
Sales and service	722 (13.1)	433 (60.0)	289 (40.0)
Manual worker	1,061 (19.2)	699 (65.9)	362 (34.1)
Labor union			
No	3,435 (62.3)	2,141 (62.3)	1,294 (37.7)
Yes	2,077 (37.7)	1,496 (72.0)	581 (28.0)
Workplace safety organization			
No	2,751 (49.9)	1,556 (56.6)	1,195 (43.4)
Yes	2,761 (50.1)	2,081 (75.4)	680 (24.6)

Values are presented as number (%).

**Table 2.** Occupational injuries among the study participants

Variables	Occupational injury		p-value
	No	Yes	
Total	5,435 (98.6)	77 (1.4)	
Sex			0.015
Male	2,949 (98.2)	53 (1.8)	
Female	2,486 (99.0)	24 (1.0)	
Age (yr)			0.482
20-29	1,043 (98.5)	16 (1.5)	
30-39	1,049 (98.2)	19 (1.8)	
40-49	1,291 (98.9)	15 (1.1)	
50-59	1,378 (98.9)	15 (1.1)	
≥60	674 (98.3)	12 (1.7)	
Education			0.153
High school or lower	853 (99.2)	7 (0.8)	
University or higher	4,582 (98.5)	70 (1.5)	
Employment			0.297
Direct	4,829 (98.7)	65 (1.3)	
Indirect	606 (98.1)	12 (1.9)	
Income			0.478
Q1 (lowest)	621 (98.4)	10 (1.6)	
Q2	3,165 (98.8)	39 (1.2)	
Q3	1,058 (98.1)	21 (1.9)	
Q4	503 (98.8)	6 (1.2)	
Q5 (highest)	88 (98.9)	1 (1.1)	
Working hours (hr/wk)			0.162
<40	3,512 (98.8)	42 (1.2)	
41-52	1,596 (98.3)	28 (1.7)	
>52	327 (97.9)	7 (2.1)	
Shiftwork			0.176
Day work	4,880 (98.7)	65 (1.3)	
Shiftwork	555 (97.9)	12 (2.1)	
Occupation			0.755
Managerial and professional	631 (98.4)	10 (1.6)	
Office worker	3,044 (98.6)	44 (1.4)	
Sales and service	715 (99.0)	7 (1.0)	
Manual worker	1,045 (98.5)	16 (1.5)	
Labor union			0.001
No	3,402 (99.0)	33 (1.0)	
Yes	2,033 (97.9)	44 (2.1)	
Workplace safety organization			0.040
No	2,722 (98.9)	29 (1.1)	
Yes	2,713 (98.3)	48 (1.7)	

Values are presented as number (%).

**Table 3.** Safety climate and the occurrence of occupational injuries

Variables	No injuries	Injuries	p-value
Total	5,435 (98.6)	77 (1.4)	
Safety climate (safety issues)			0.006
Favorable (<2)	3,598 (98.9)	39 (1.1)	
Unfavorable (≥ 2)	1,837 (98.0)	38 (2.0)	
Components of safety climate			
Management ensures that everyone receives the necessary information on safety			0.152
Yes	3,963 (98.8)	50 (1.2)	
No	1,472 (98.2)	27 (1.8)	
Management encourages employees to work in accordance with safety rules, even when the work schedule is tight			0.019
Yes	3,938 (98.8)	46 (1.2)	
No	1,497 (98.0)	31 (2.0)	
Management involves employees in decisions regarding safety			0.204
Yes	3,520 (98.8)	44 (1.2)	
No	1,915 (98.3)	33 (1.7)	
We help each other work safely			0.033
Yes	4,376 (98.8)	54 (1.2)	
No	1,059 (97.9)	23 (2.1)	
We consider minor accidents a normal part of our daily work			0.153
No	3,699 (98.8)	46 (1.2)	
Yes	1,736 (98.2)	31 (1.8)	

Values are presented as number (%).

**Table 4.** Associations between occupational injuries and safety climate by multiple logistic regression analysis^1^

Factors	Model 1	Model 2	Model 3
Safety climate (safety issues)			
Favorable (<2)	1.00 (reference)	1.00 (reference)	1.00 (reference)
Unfavorable (≥ 2)	1.93 (1.22, 3.04)	1.91 (1.21, 3.02)	2.20 (1.38, 3.51)
Component of safety climate			
Management does not ensure that everyone receives the necessary information on safety	1.49 (0.92, 2.38)	1.48 (0.91, 2.37)	1.83 (1.10, 2.98)
Management does not encourage employees to work in accordance with safety rules, even when the work schedule is tight	1.79 (1.11, 2.82)	1.75 (1.09, 2.79)	2.02 (1.25, 3.24)
Management does not involve employees in decisions regarding safety	1.41 (0.88, 2.22)	1.40 (0.88, 2.21)	1.56 (0.97, 2.49)
We do not help each other work safely	1.80 (1.08, 2.93)	1.77 (1.05, 2.88)	1.98 (1.17, 3.25)
We consider minor accidents a normal part of our daily work	1.39 (0.86, 2.20)	1.35 (0.84, 2.14)	1.35 (0.84, 2.14)

Values are presented adjusted odds ratio (95% confidence interval).

1 Model 1: Adjusted for sex and age; Model 2: Adjusted for sex, age, income, working hours, occupation, and employment status; Model 3: Adjusted for sex, age, income, working hours, occupation, employment status, and the presence of labor unions and workplace safety organizations.

## Supplementary materials

Supplementary Material 1. Presence of labor union and the safety climate of the workplace

Supplementary Material 2. Multiple logistic regression analysis of the correlation between occupational injuries and safety climate stratified by occupation group
