# Supplementary material for: Association between the safety climate and occupational injury in the Korean working population: a cross-sectional study
Source: Epidemiol Health. 2024 Oct 1;46:e2024082. doi: 10.4178/epih.e2024082 (PMC11832241; doi:10.4178/epih.e2024082)
Supplement: Supplementary Material 1. — Presence of labor union and the safety climate of the workplace [file epih-46-e2024082-Supplementary-1.docx]

Supplementary Material 1. Presence of labor union and the safety climate of the workplace

|  | **Labor union** | |  |
| --- | --- | --- | --- |
|  | **No (n=3435)** | **Yes (n=2077)** | **p-value** |
| **Safety climate** |  |  | **<0.0001** |
| Favorable (<2 safety issues) | 2445 (71.2) | 1683 (81.0%) |  |
| Unfavorable (≥ 2 safety issues) | 990 (28.8%) | 394 (19.0%) |  |
| **Component of safety climate** |  |  |  |
| **Management ensures that everyone receives the necessary information on safety** |  |  | **<0.0001** |
| Yes | 2329 (67.8%) | 1684 (81.1%) |  |
| No | 1106 (32.2%) | 393 (18.9%) |  |
| **Management encourages employees to work in accordance with safety** |  |  | **<0.0001** |
| **rules, even when the work schedule is tight** |  |  |  |
| Yes | 2364 (68.8%) | 1620 (78.0%) |  |
| No | 1071 (31.2%) | 457 (22.0%) |  |
| **Management involves employees in decisions regarding safety** |  |  | **<0.0001** |
| Yes | 2105 (61.3%) | 1459 (70.2%) |  |
| No | 1330 (38.7%) | 618 (29.8%) |  |
| **We help each other work safely** |  |  | **<0.0001** |
| Yes | 2683 (78.1%) | 1747 (84.1%) |  |
| No | 752 (21.9%) | 330 (15.9%) |  |
| **We consider minor accidents a normal part of our daily work** |  |  | 0.241 |
| No | 2354 (68.5%) | 1391 (67.0%) |  |
| Yes | 1081 (31.5%) | 686 (33.0%) |  |
