# Supplementary material for: Association between the safety climate and occupational injury in the Korean working population: a cross-sectional study
Source: Epidemiol Health. 2024 Oct 1;46:e2024082. doi: 10.4178/epih.e2024082 (PMC11832241; doi:10.4178/epih.e2024082)
Supplement: Supplementary Material 2. — Multiple logistic regression analysis of the correlation between occupational injuries and safety climate stratified by occupation group [file epih-46-e2024082-Supplementary-2.docx]

**Supplementary Material 2. Multiple logistic regression analysis of the correlation between occupational injuries and safety climate stratified by occupation group.**

|  | **White collar** | **Blue collar** | **Pink Collar** |
| --- | --- | --- | --- |
| **Factor** | **OR (95% CI)** | **OR (95% CI)** | **OR (95% CI)** |
| **Safety Climate** |  |  |  |
| Favorable (Problems <2) | Reference | Reference | Reference |
| Unfavorable (Problems ≥ 2) | **2.85 (1.63–5.03)** | 1.20 (0.38–3.46) | 1.62 (0.29–8.03) |
| **Problems of Safety climate** |  |  |  |
| Management DO NOT ensure that everyone receives the necessary information on safety | **2.23 (1.23–3.96)** | 0.99 (0.26–3.21) | 1.20 (0.16–6.14) |
| Management DO NOT encourage employees to work in accordance with safety  rules-even when the work schedule is tight | **2.56 (1.45–4.50)** | 0.89 (0.24–2.75) | 2.27 (0.41–11.42) |
| Management DO NOT involve employees in the decisions regarding safety | **2.31 (1.32–4.05)** | 0.45 (0.12–1.43) | 0.77 (0.11– 3.87) |
| We DO NOT help each other to work safely | **2.90 (1.59–5.14)** | 0.56 (0.09–2.12) | 0.89 (0.04–6.01) |
| We work with considering minor accidents as a normal part of our daily work | 1.28 (0.72–2.23) | 2.10 (0.71–6.08) | 1.46 (0.27–6.99) |

CI, confidence interval; OR, odds ratio

Model : Adjusted for sex, age group, income, working hour, presence of labor union, and workplace safety organization
